# Design and Preliminary Findings of Adherence to the Self-Testing for Our Protection From COVID-19 (STOP COVID-19) Risk-Based Testing Protocol: Prospective Digital Study

**DOI:** 10.2196/38113

**Published:** 2022-06-16

**Authors:** Carly Herbert, Vik Kheterpal, Thejas Suvarna, John Broach, Juan Luis Marquez, Ben Gerber, Summer Schrader, Christopher Nowak, Emma Harman, William Heetderks, Nisha Fahey, Elizabeth Orvek, Peter Lazar, Julia Ferranto, Kamran Noorishirazi, Shivakumar Valpady, Qiming Shi, Honghuang Lin, Kathryn Marvel, Laura Gibson, Bruce Barton, Stephenie Lemon, Nathaniel Hafer, David McManus, Apurv Soni

**Affiliations:** 1 Program in Digital Medicine Department of Medicine University of Massachusetts Chan Medical School Worcester, MA United States; 2 CareEvolution, Inc Ann Arbor, MI United States; 3 Department of Emergency Medicine University of Massachusetts Chan Medical School Worcester, MA United States; 4 Washtenaw County Health Department Washtenaw, MI United States; 5 Department of Population and Quantitative Health Sciences University of Massachusetts Chan Medical School Worcester, MA United States; 6 National Institute of Biomedical Imaging and Bioengineering National Institutes of Health Kelly Services Bethesda, MD United States; 7 Department of Pediatrics University of Massachusetts Chan Medical School Worcester, MA United States; 8 University of Massachusetts Center for Clinical and Translational Science University of Massachusetts Chan Medical School Worcester, MA United States; 9 Division of Clinical Informatics Department of Medicine University of Massachusetts Chan Medical School Worcester, MA United States; 10 Division of Infectious Disease Department of Medicine University of Massachusetts Chan Medical School Worcester, MA United States; 11 Division of Cardiology Department of Medicine University of Massachusetts Chan Medical School Worcester, MA United States

**Keywords:** COVID-19, rapid antigen tests, COVID-19 testing, infectious disease, disease spread, prevention, coronavirus, adherence, reporting, mHealth, health application, mobile health, digital health, public health, surveillance, health care, smartphone app, vaccination, digital surveillance

## Abstract

**Background:**

Serial testing for SARS-CoV-2 is recommended to reduce spread of the virus; however, little is known about adherence to recommended testing schedules and reporting practices to health departments.

**Objective:**

The Self-Testing for Our Protection from COVID-19 (STOP COVID-19) study aims to examine adherence to a risk-based COVID-19 testing strategy using rapid antigen tests and reporting of test results to health departments.

**Methods:**

STOP COVID-19 is a 12-week digital study, facilitated using a smartphone app for testing assistance and reporting. We are recruiting 20,000 participants throughout the United States. Participants are stratified into high- and low-risk groups based on history of COVID-19 infection and vaccination status. High-risk participants are instructed to perform twice-weekly testing for COVID-19 using rapid antigen tests, while low-risk participants test only in the case of symptoms or exposure to COVID-19. All participants complete COVID-19 surveillance surveys, and rapid antigen results are recorded within the smartphone app. Primary outcomes include participant adherence to a risk-based serial testing protocol and percentage of rapid tests reported to health departments.

**Results:**

As of February 2022, 3496 participants have enrolled, including 1083 high-risk participants. Out of 13,730 tests completed, participants have reported 13,480 (98.18%, 95% CI 97.9%-98.4%) results to state public health departments with full personal identifying information or anonymously. Among 622 high-risk participants who finished the study period, 35.9% showed high adherence to the study testing protocol. Participants with high adherence reported a higher percentage of test results to the state health department with full identifying information than those in the moderate- or low-adherence groups (high: 71.7%, 95% CI 70.3%-73.1%; moderate: 68.3%, 95% CI 66.0%-70.5%; low: 63.1%, 59.5%-66.6%).

**Conclusions:**

Preliminary results from the STOP COVID-19 study provide important insights into rapid antigen test reporting and usage, and can thus inform the use of rapid testing interventions for COVID-19 surveillance.

## Introduction

Despite relatively widespread vaccination for SARS-CoV-2 throughout the United States, in late February 2022, nearly 90,000 new cases of COVID-19 were reported daily in the United States among both unvaccinated and vaccinated individuals, and herd immunity remains uncertain [1]. With relaxation of masking and social distancing requirements, and many US residents returning to in-person work and schooling, widespread, accessible testing for COVID-19 is an integral component of the federal strategy to safely establish a “new normal” [2-4].

Antigen detection rapid diagnostic tests (Ag-RDTs) for COVID-19 pose great opportunity for community surveillance owing to their relative ease of use and quick turn-around time for results, making them amenable to testing outside traditional clinical environments [5]. Serial testing 2-3 times per week with Ag-RDTs is recommended to detect SARS-CoV-2 infections, specifically asymptomatic infections that comprise over 50% of total infections [6,7]. Despite the availability of this effective testing approach, little is known about adherence to this schedule outside of a controlled trial environment [8,9]. Additionally, it is unknown how COVID-19 testing strategies and schedules should be optimized based on risk factors for infection, including vaccination status [4,10,11]. At-home Ag-RDTs for COVID-19 may also challenge public health surveillance efforts owing to their reliance on individual users to carry out the tests appropriately and report their test results. Indeed, when Ag-RDTs were streamlined through the Food and Drug Administration authorization process in spring 2021, this resulted in device launches without systematic reporting mechanisms in place, leaving large potential gaps in COVID-19 surveillance data [12,13]. Public health reporting practices at the individual and practitioner levels are unknown, although likely highly varied, which present challenges in interpreting current public health data.

To fill these knowledge gaps, we are performing a longitudinal study to examine adherence to a risk-based testing protocol supported by a digital infrastructure to allow recruitment and study engagement nationwide. The goals of this prospective, site-less digital study are to leverage strong partnerships with community organizations and local health departments to assess adherence to a risk-based SARS-CoV-2 testing strategy using over-the-counter tests, and to describe participants’ behavior for reporting test results to public health departments and factors associated with test reporting behavior.

## Methods

### Study Population and Recruitment

We are recruiting up to 20,000 participants throughout the United States who meet our predefined inclusion/exclusion criteria (Textbox 1). Study enrollment is taking place in two phases: phase 1 enrollment is restricted to Michigan residents using a convenience sample, leveraging momentum and community connections from previous COVID-19 interventions; phase 2 enrollment is open to participants anywhere in the continental United States. Recruitment efforts are being spearheaded by the RADx Community Health Equity and Engagement Team [14], with the goal of recruiting a geographic, racially, and ethnically diverse sample across the United States. Phase 2 recruitment includes respondent-driven sampling, stratified sampling, and use of digital access codes to improve representation of diverse populations in our cohort. The research team also recruits through targeted social media outreach, word of mouth, community networks, and direct communications with the support of community partners. Community partners, including community organizations and local and state health departments, are identified through the professional networks of the study team members. These partners are intentionally selected based upon their ability to serve and reach large numbers of individuals who are diverse with respect to socioeconomic status and race/ethnicity. Representatives of these organizations are sent an email description of the study and asked to distribute it through their listservs. These emails also include flyers that can be posted in community locations. The recruitment strategy is adjusted throughout the study to identify populations from regions where there is an outbreak of COVID-19 or where vaccination rates are relatively low.

The Self-Testing for Our Protection from COVID-19 (STOP COVID-19) study utilizes the Mstudy app, a custom smartphone app created within the MyDataHelps interface. The app is used to electronically collect survey data from all participants, as well as guide participants through rapid antigen testing and interpreting their results. Residents of the mainland United States with the MyDataHelps app receive an in-app notification inviting them to participate in the study. Community partners distribute emails and flyers to interested participants with instructions to download the Mstudy app and enter a join code for the study; participants are also able to autonomously sign up through the study website. The Mstudy app is free of charge and compatible with both Apple and Android smartphone devices. All participants consent and enroll in the study using the Mstudy app. Participants less than 18 years old are required to assent, as well as receive written consent from their parents/guardians. On enrollment, participants’ street address is also collected and verified against the United States Postal Service database through the digital platform to ensure that study staff can ship required testing materials directly to participants’ homes. Participants are eligible for a US $50 gift card on two separate occasions throughout the study, based on their completion of surveys and rapid antigen tests.

**Inclusion and exclusion criteria of the STOP COVID-19 study.**

**Inclusion criteria**

- ≥8 years of age
- Access to a smartphone
- Speak English or Spanish
- Able to provide informed consent or assent with parental consent (for participants under 18 years old)

**Exclusion criteria**

- Current incarceration
- Lack of mailing address
- Lack smartphone internet access
- Living outside mainland United States

### Wearable Data Collection

After enrollment, participants are asked if they would like to securely and confidentially share information from their wearable activity-tracker device (ie Fitbit, Apple Watch, and Google Fit). If participants consent to sharing wearable data, data from their activity tracker are passively collected for the 3-month study period, with no additional requirements from the participant. Sharing wearable data is optional, and participants may decline to share wearable data and still participate fully in the study. The study platform is enabled to collect measures of physical activity (eg, daily steps, distance walking or running, stairs climbed, standing time), mobility (eg, walking speed, walking asymmetry percentage, step length), vitals (eg, heart rate, body temperature, respiratory rate, resting heart rate, heart rate variability, and oxygen saturation), and sleep analysis, depending on the type of wearable device activated.

### Risk Stratification

**Figure 1:**
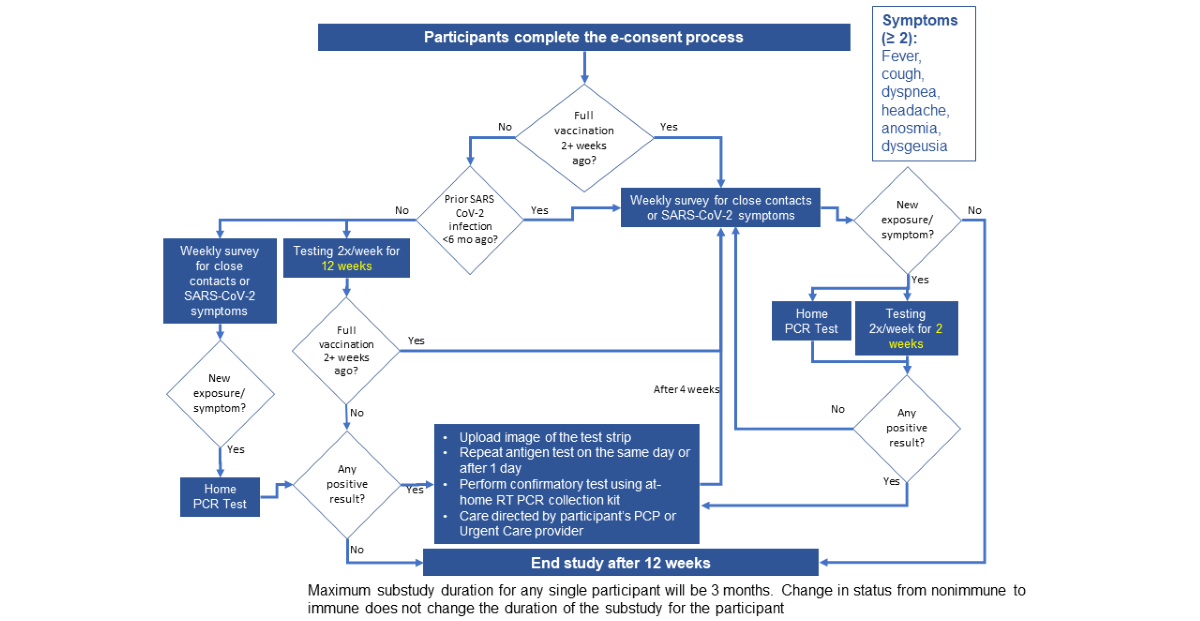
STOP COVID-19 risk stratification process and testing schedule. mo: months; PCP: primary care provider; PCR: polymerase chain reaction; RT PCR: real-time polymerase chain reaction.

On enrollment in the study, participants are stratified into high- or low-risk groups based on SARS-CoV-2 vaccination and infection history (Figure 1, Multimedia Appendix 1). High-risk participants are defined as those who are not fully vaccinated for SARS-CoV-2, as defined by Centers for Disease Control and Prevention (CDC) guidelines, and have not had an infection in the past 6 months [15]. Low-risk participants are those who have been fully vaccinated for SARS-CoV-2 and/or infected with COVID-19 in the past 6 months.

### High-Risk Participant Study Procedures

High-risk participants are asked to test for COVID-19 twice per week for 12 consecutive weeks using an Ag-RDT (Quidel QuickVue) with the Mstudy app for testing assistance. High-risk participants receive a weekly surveillance questionnaire to assess if they are experiencing two or more new COVID-19–related symptoms (eg, fever or chills, shortness of breath, cough, loss of taste, and headache) or have had close contact with someone who tested positive for SARS-CoV-2 in the past 7 days. In the case of two or more symptoms or exposure, participants are sent a home polymerase chain reaction (PCR) collection kit via Quest Diagnostics and told to continue using the Ag-RDT at home, as the PCR test is the current diagnostic gold standard. The home PCR kit should be utilized immediately upon receipt to collect a nasal specimen and returned to Quest in the preaddressed envelope within 24 hours. Participants receive all testing and survey reminders through the app.

### Low-Risk Participant Study Procedures

Low-risk participants are asked to complete weekly surveillance questionnaires to monitor for COVID-19–related symptoms or exposure to SARS-CoV-2. If a low-risk participant reports two or more COVID-19–related symptoms and/or a close exposure to someone with SARS-CoV-2, they are sent Ag-RDT kits within 48 hours and advised to test twice weekly for 2 weeks following the symptom or exposure. Additionally, participants are sent a home PCR collection kit and asked to self-collect a nasal specimen and ship it back to Quest Laboratories using the preaddressed envelope within 24 hours. After completing the 2-week testing period, low-risk participants resume completing weekly surveillance questionnaires.

### Testing and Reporting Results

The Mstudy app contains detailed instructions for using the rapid tests for COVID-19 (Multimedia Appendix 1). Participants are instructed on how to correctly swab the nasal cavity and utilize the testing equipment. The app includes a timer that alerts the participant when the test is ready to be read and walks the participant through how to interpret the test results (ie, positive, negative, or inconclusive). Within the Mstudy app, participants are asked to provide an interpretation of their test result and to upload a picture of the test into the app. All positive tests are confirmed by study coordinators. If participants test positive for SARS-CoV-2, they are contacted by a physician associated with the study and advised to follow CDC guidelines for self-isolation, as well as to seek care from their primary care provider if needed. Each time a participant records their test results, they are asked if they would like to opt-in for automated reporting, either in a full or deidentified manner. Results of tests from users who agree to report either fully or anonymously are sent to a federal system (“Report Stream”) through the study mobile app (Multimedia Appendix 2, Figure S1). Results of tests from users who agree to report full (identified) information will also be sent to their respective state health department. The app is enabled to report results to all state departments of health. All reporting is done through the Mstudy app, with no additional user burden. Participants can adjust their preference for reporting test results at any time point using the app.

### Questionnaire Schedule

Study participants complete weekly surveillance questionnaires through the Mstudy app, as well as additional surveys during enrollment, at baseline, after each at-home test, and on conclusion of the 3-month study period (Multimedia Appendix 2, Table S1). In addition to testing information, weekly surveillance, and information used to determine risk stratification (prior infection and vaccination), surveys gather data on participants’ demographic characteristics, COVID-19 beliefs and risk perceptions, health care utilization, medical history and health status, and reporting attitudes and perceptions. Each survey takes 2-15 minutes to complete. Participants receive reminders to complete assigned surveys through the Mstudy app.

### Data Management

All testing and questionnaire data are securely stored within rkStudio, the management platform of the Mstudy app. All collected data are deidentified using participant IDs prior to analysis and stored in the secure University of Massachusetts Chan Medical School server. PCR test results are connected to Mstudy data by participant ID.

### Ethics Approval

This study protocol was approved by the Institutional Review Board of the University of Massachusetts Chan Medical School and externally by the Western Institutional Review Board-Copernicus Group (now named WCG; the Institutional Review Board number is 20213392.

### Analytical Plan

Primary outcome variables of the study include adherence to the risk-based SARS-CoV-2 testing strategy, and test result reporting behaviors and motivations to participants’ respective state department of health. In prespecified analyses, we will identify patterns of testing adherence over time, and identify factors associated with decreased testing adherence. We will also perform an ordinal logistic regression to assess whether participants’ behavior for reporting test results to the department of health is associated with test result, vaccination status, infection history, or sociodemographic and psychosocial variables. Lastly, incidence rates of COVID-19 for participants under surveillance will be calculated overall during the study period and separately as person-time during the testing period with stratification according to participants’ risk status. Risk of infection among low-risk participants will be estimated in relation to time since vaccination or infection using time-to-event analyses.

In the preliminary analysis presented, demographics from phase 1 of the study were tabulated by risk category. Testing adherence was measured among high-risk participants who finished the 12-week testing period. Adherence to the testing schedule was determined on a weekly basis, and participants were considered “adherent” to the schedule if they tested two times each week. Adherence was categorized into no, low, moderate, and high adherence groupings. The “no adherence” group included individuals who were never adherent to the testing schedule during the study period. The “low adherence” group included individuals who were adherent for 1 to 4 weeks of the 12-week study period. “Moderate adherence” included those who were adherent for 5 to 8 weeks of the study period, and “high adherence” was defined as testing twice weekly for 9 or more weeks of the study. Reporting choices were calculated at the participant level to avoid overrepresenting the reporting choices of frequent testers. Reporting choices were tabulated by risk category, test result, and adherence category, and 95% CIs were calculated using the Clopper-Pearson interval.

## Results

### Enrollment

**Figure 2:**
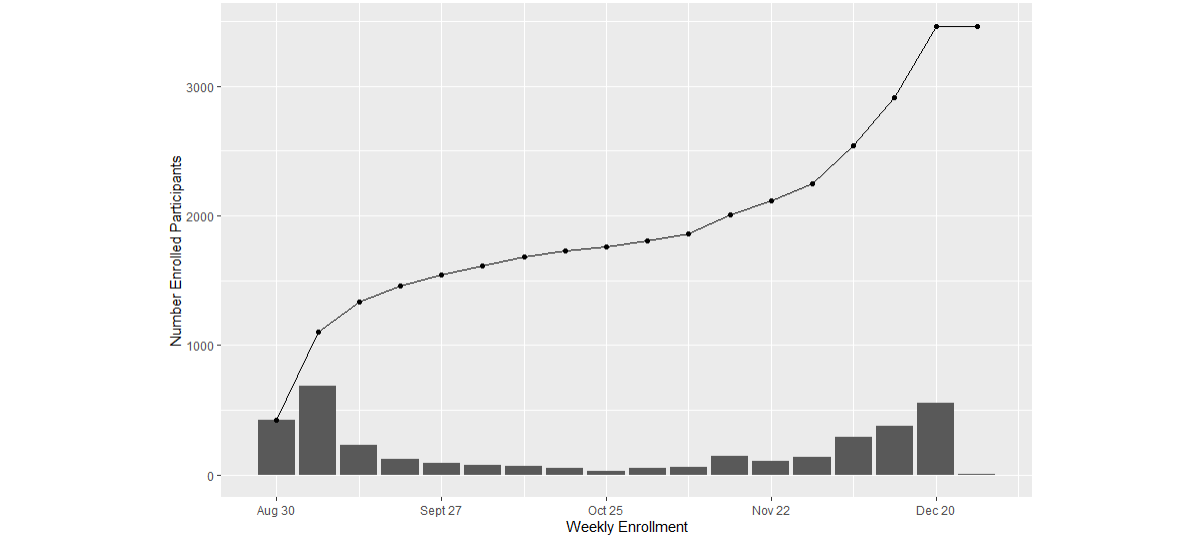
STOP COVID-19 enrollment, fall 2021.

The STOP COVID-19 study began enrolling phase-1 participants in August 2021, and 3496 participants have enrolled in the study to date, including 1083 high-risk participants (Figure 2 and Figure S2 in Multimedia Appendix 2). Phase 2 of recruitment started in February 2022 and is ongoing. Women make up the majority of both low- and high-risk participants, and approximately 20% of high-risk participants are children under 18 years old (Table 1). Among the adult participants, approximately 80% have a bachelor’s degree or higher. Most participants are White, with Asian participants making up 8.8% and 5.0% of low- and high-risk participants, respectively (Table 1). Only approximately 1% of participants are over the age of 75 years. High-risk participants with symptoms and those with both symptoms and known exposure have a 3.66- and 1.39-times higher positivity rate for COVID-19 than low-risk participants, respectively.

**Table 1:** Demographic characteristics of STOP COVID-19 participants.

Characteristics		High-risk participants (n=1083)	Low-risk participants (n=2413)
**Age (years), n (%)**			
	8-17	202 (18.65)	135 (5.59)
	18-30	106 (9.79)	423 (17.53)
	31-45	375 (34.63)	1072 (44.42)
	46-60	214 (19.76)	500 (20.72)
	61-75	145 (13.39)	237 (9.82)
	>75	19 (1.75)	22 (0.91)
	Missing	22 (2.03)	24 (0.95)
**Gender, n (%)**			
	Man	314 (28.99)	596 (24.70)
	Woman	660 (60.94)	1564 (64.82)
	Transgender	3 (0.28)	9 (0.37)
	Nonbinary	13 (1.20)	46 (1.90)
	Missing	93 (8.59)	198 (8.21)
**Race, n (%)**			
	White	864 (79.78)	1819 (75.38)
	Asian	54 (4.99)	212 (8.79)
	Black/African-American	22 (2.03)	50 (2.07)
	Native American/Alaskan Native	0 (0)	1 (0.04)
	Native Hawaiian or Pacific Islander	0 (0)	2 (0.08)
	Multiracial	32 (2.95)	71 (2.94)
	Other	9 (0.83)	36 (1.49)
	Missing	102 (9.42)	222 (9.20)
**Hispanic, n (%)**			
	Yes	52 (4.80)	93 (3.85)
	No	936 (86.43)	2108 (87.36)
	Missing	95 (8.77)	212 (8.79)
**Education level, n (%)**			
	Bachelor’s degree or higher	654 (60.39)	1750 (72.52)
	Some college	145 (13.39)	265 (10.98)
	High school graduate	33 (3.05)	80 (3.32)
	Did not finish high school	134 (12.37)	110 (4.56)
	Missing	117 (10.80)	208 (8.62)
**Employment status, n (%)**			
	Working now, permanently	584 (53.92)	1398 (57.94)
	Working now, temporarily	29 (2.68)	107 (4.43)
	Student	190 (17.54)	332 (13.76)
	Retired	96 (8.86)	146 (6.05)
	Keeping house	50 (4.62)	126 (5.22)
	Unemployed	21 (1.94)	77 (3.19)
	Missing	113 (10.43)	227 (9.41)
**Positivity of Ag-RDT^a^ tests, % (95% CI)**			
	Total	7.5 (5.9-9.4)	11.0 (8.8-13.5)
	Symptomatic	14.3 (8.0-22.8)	3.9 (1.7-7.5)
	Close contact exposure	5.2 (2.7-8.9)	6.9 (4.3-10.5)
	Both symptoms and exposure	31.1 (20.8-42.9)	22.3 (17.1-28.2)
	Neither	3.9 (2.4-5.9)	N/A^b^
**Test reporting decisions, % (95% CI)^c^**			
	Full reporting	67.4 (64.4-70.3)	66.9 (63.5-70.2)
	Anonymous reporting	28.2 (25.5-31.1)	30.1 (26.9-33.4)
	No reporting	4.3 (3.2-5.8)	3.0 (1.9-4.4)

^a^Ag-RDT: antigen rapid diagnostic test.

^b^N/A: not applicable; low-risk participants were only eligible for testing after reporting symptoms or close contact exposure.

^c^This sample only included participants who recorded at least one test result in the Mstudy app: n=1016 for the high-risk group and n=801 for the low-risk group.

### Test Reporting

As of February 2022, participants have completed over 13,730 rapid tests and have reported 13,480 (98.2%, 95% CI 97.9%-98.4%) results to their respective state public health departments. Reporting differed by test result (positive, negative, or invalid), with 3.7% (95% CI 2.0%-6.1%) of positive tests unreported in comparison to 1.8% (95% CI 1.5%-2.0%) of negative tests reported. Reporting choices did not differ significantly by risk category (Table 1). Among those who have chosen not to report their test results, the most cited reason is not wanting to be contacted by the government. Other reasons include not trusting the government, not knowing how to report, believing reporting is not useful, and being worried about missing work.

### Testing Adherence and Impact on Reporting

Twelve weeks of adherence to twice-weekly serial testing was assessed among 622 high-risk participants who completed the full study period. Of these participants, 223 (35.9%) were highly adherent to the testing protocol (Table 2). The percentage of tests reported with full personal identifiers to the state department of health was significantly higher among those with high adherence, as compared to moderate and low adherent participants (Table 2). Nonreporting was significantly higher among participants with moderate adherence in comparison to those with high adherence; however, nonreporting did not differ between participants with low and high adherence (Table 2).

**Table 2:** Reporting decisions per test by adherence group among high-risk participants (N=622).

Variable		No adherence	Low adherence	Moderate adherence	High adherence	Total
Participants, n (%)		103 (16.6)	162 (26.0)	134 (21.5)	223 (35.9)	622 (100.0)
Total tests completed, n		13	729	1693	4145	6580
**Reporting, % (95% CI)**						
	Full reporting	53.9 (25.1-80.8)	63.1 (59.5-66.6)	68.3 (66.0-70.5)	71.7 (70.3-73.1)	69.8 (68.7-70.9)
	Anonymous reporting	30.8 (9.1-61.4)	34.8 (31.4-38.4)	28.1 (26.0-30.3)	26.5 (25.2-27.9)	27.9 (26.8-29.0)
	No reporting	15.4 (1.9-45.4)	2.1 (1.2-3.4)	3.6 (2.8-4.6)	1.8 (1.4-2.2)	2.3 (2.0-2.7)

## Discussion

### Principal Findings

The STOP COVID-19 study is a novel longitudinal, digital study aimed at understanding adherence and public health reporting of rapid antigen testing throughout the United States, using a risk-based testing protocol. Here, we describe the study methodology, which utilizes the Mstudy app for data collection and study coordination purposes, and preliminary results from phase 1 of participant recruitment in Michigan. Phase 1 of enrollment began in August 2021, and 3496 participants in Michigan have enrolled to date, including more than 1000 high-risk participants. Digital site-less studies have many advantages in the age of COVID-19, especially to facilitate study recruitment and retention. The site-less study approach has allowed us to dynamically change the recruitment strategy throughout the study to prioritize communities with high prevalence of SARS-CoV-2, seasonal surges, or low vaccination rates in order to optimally sample communities with a high burden of COVID-19. Further, digital studies require less active study coordination than traditional site-based studies because of the ability of technology to facilitate certain tasks (ie, consenting and providing instructions for study-related activities), which allowed us to implement a risk-based testing algorithm in participants’ homes nationwide while conserving the time and resources of study personnel. The data collected from this study will offer tremendous insight into COVID-19 at-home testing behaviors, and using the digital approach was highly effective.

### Adherence and Motivations for Serial Rapid Testing

There has been significant interest in rapid antigen testing on a national level as a cost-effective solution to expand serial testing for COVID-19 for surveillance and asymptomatic case detection. However, little is known about perceptions of serial testing and how individuals use rapid test results in altering their COVID-19 risk behaviors. Among the study participants engaged in rapid serial testing, the COVID-19 positivity rate was the highest among high-risk, exposed, symptomatic participants, consistent with the published literature, and supporting our approach to risk stratification [16]. Based on our preliminary data, only 35.9% of high-risk participants displayed high adherence to the weekly serial testing schedule during the study period. Although qualitative studies have identified motives for frequent testing, including a fear of unknowingly spreading COVID-19 to others, this is the first study to quantitatively evaluate adherence to serial testing programs [17]. Further, no previous studies have used a longitudinal risk-based approach to testing, which has been suggested as a key strategy in establishing a “new normal” in a world burdened by COVID-19 [11].

### Perceptions of Public Health Reporting

Throughout the COVID-19 pandemic, perceptions and trust of government have strongly influenced the adoption or rejection of public health initiatives, including masking, social distancing, and vaccination. Our preliminary results showed that 14,000 rapid tests have been completed by study participants, with a full or anonymized reporting rate to participants’ respective state department of public health of over 98%. We observed statistically significant differences in reporting status based on test result, with negative results being reported more than positive results.

In a study of 1420 Australian adults, individuals with higher trust in government had 6-times greater odds of adopting recommended COVID-19 avoidance behaviors, including social distancing, self-quarantine, and hand washing, than individuals with low governmental trust [18]. Additionally, high public trust in the government has been found to favorably impact the use of COVID-19 control measures, as well as increase the likelihood of vaccination for SARS-CoV-2 [19-22]. Government trust has also been highly associated with an individual’s demographic characteristics and social network. A study from 178 countries found that public trust in the government during COVID-19 was positively associated with older age and good health, and negatively associated with higher education [23]. Further, we found that highly adherent participants reported a higher proportion of results to their state department of health with full identifying information than participants with moderate or low adherence to the STOP COVID-19 testing schedule. This supports the notion that COVID-19 protective (preventative) behaviors are clustered among certain individuals [24]. These results will help to shape public health messaging and initiatives, especially as rapid at-home testing becomes more accessible and widespread.

### Site-Less Study Implementation Challenges

The site-less digital study approach is still relatively new in the fields of medical and public health research, although it has garnered significant interest owing to the potential benefits to participant accessibility, engagement, longitudinal data volume, and study administrative costs [25]. As the pandemic changes in severity by location and time, we believe the site-less study design is uniquely suited to provide insight into numerous communities to observe how testing behaviors change over time and vary throughout the country. In addition to fully consenting participants and collecting data through a mobile app, this study ships participants’ tests for COVID-19 directly to their homes in a continuous fashion over 3 months, based on their risk category, exposures, and symptoms, which has resulted in notable implementation challenges. Although the process for rapid test distribution has been streamlined within the Mstudy app, with kit orders placed immediately based on survey responses, the PCR ordering process through Quest has been more complex. Initially, when a participant reported COVID-19 symptoms or exposure and qualified for PCR testing, they were prompted within the app to follow a link to the Quest website to register for a PCR test kit. This process was confusing and time-consuming for participants, and study coordinators were tasked with calling all eligible participants to explain the process. Further, participant registration often resulted in key identifiers being omitted from test orders and inability to match PCR test results to data from the Mstudy app. In the past 2 months, our team has worked closely with Quest to implement a roster system, by which study coordinators order PCR kits on behalf of eligible participants. This has taken significant burden off the participants and has resulted in higher participant PCR testing adherence (data not shown). As we begin phase 2 enrollment, we will continue discussions to optimize test distribution to participants, as well as other issues that arise.

Additionally, during phase 1 enrollment, convenience sampling resulted in a sample of predominately highly educated, White, female participants. Further, only 1% of the phase 1 cohort is over 75 years old, despite older adults facing the most devastating outcomes due to SARS-CoV-2 [26]. We recognize it is especially important to understand the usage of SARS-CoV-2 diagnostics and mobile health tools among older adults. During phase 2 enrollment, we will be adjusting our recruitment and sampling approaches, as detailed in the Methods section, with the goal of diversifying the cohort in terms of age, race, gender, education, and geographic location.

### Study Strengths and Limitations

This is the first study to examine the reporting of COVID-19 results from Ag-RDTs and adherence to serial testing schedules without supervision, providing important data to guide the administration and use of Ag-RDTs in a real-world setting. The site-less study design offers great flexibility to recruit based on fluctuations in the pandemic, as well as to obtain a geographically diverse participant pool. Study strengths also include the use of the MyDataHelps app as a tool for reporting test results, as well as a risk-based approach to serial testing. Nevertheless, certain inclusion criteria such as the use of a smartphone may limit the generalizability of this study. Participation, nonresponse, and attrition biases are also of concern, as individuals choosing to enroll in and comply with a longitudinal study to test for COVID-19 may differ from nonparticipants in relation to COVID-19 perceptions, education, race, and health literacy.

### Conclusions

This report describes the study design and preliminary results of a longitudinal study aimed to understand individuals’ testing and reporting decisions. Site-less study designs are increasingly valuable in the era of COVID-19. The data collected in this study will provide important insights into how individuals navigate their testing decisions during the COVID-19 pandemic, as well as how we should understand the data reported from rapid antigen tests for COVID-19.

## Appendix

Multimedia Appendix 1 Screenshots of Mstudy app enrollment and testing processes.

Multimedia Appendix 2 Supplemental tables and figures.

## Abbreviations

Ag-RDT: antigen rapid diagnostic test
CDC: Centers for Disease Control and Prevention
PCR: polymerase chain reaction
STOP COVID-19: Self-Testing for Our Protection from COVID-19
