# Supplementary figures and images for: Design and Preliminary Findings of Adherence to the Self-Testing for Our Protection From COVID-19 (STOP COVID-19) Risk-Based Testing Protocol: Prospective Digital Study
Source: JMIR Form Res. 2022 Jun 16;6(6):e38113. doi: 10.2196/38113 (PMC9205422; doi:10.2196/38113)

## Slide 1
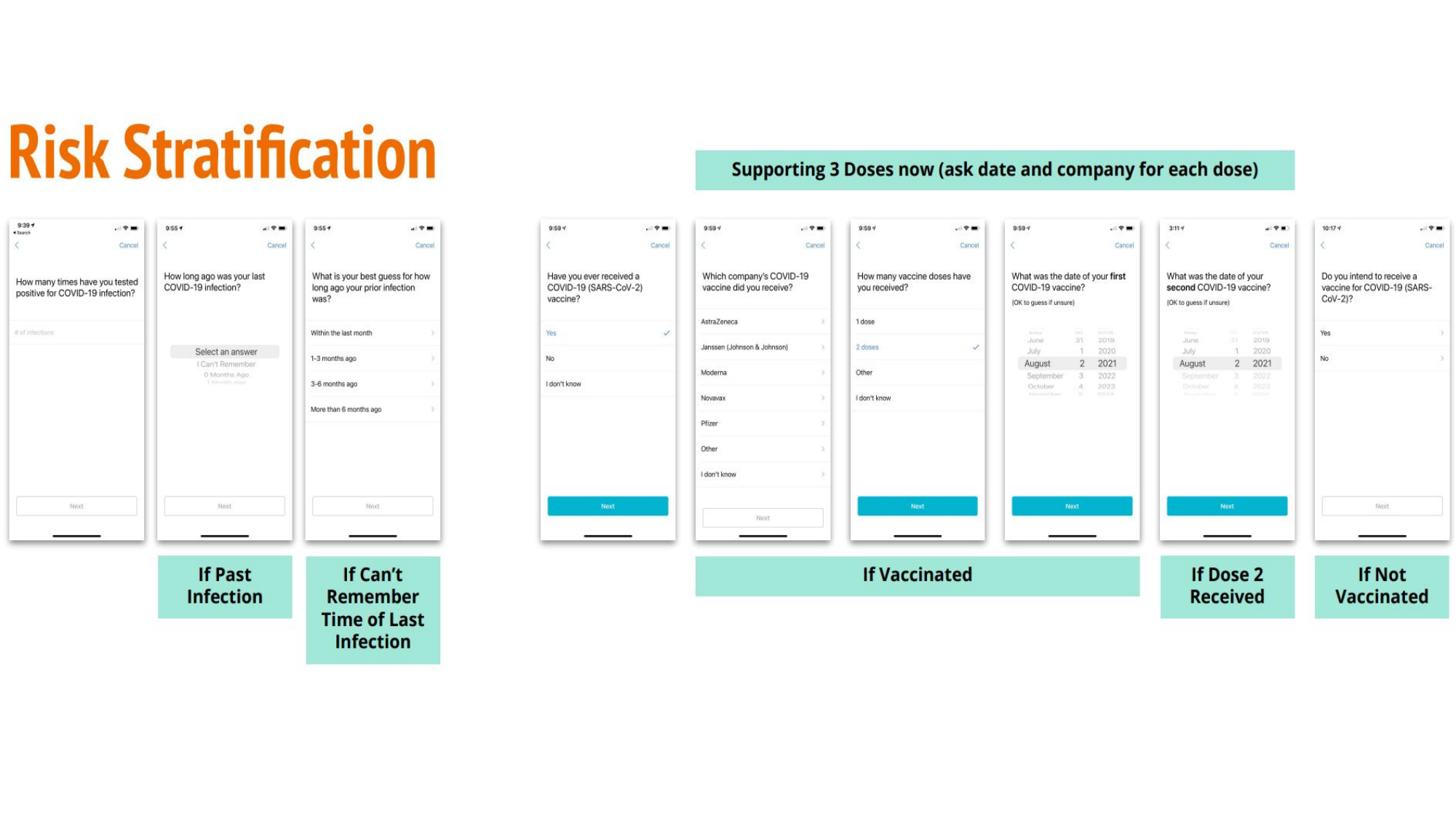

#

## Slide 2
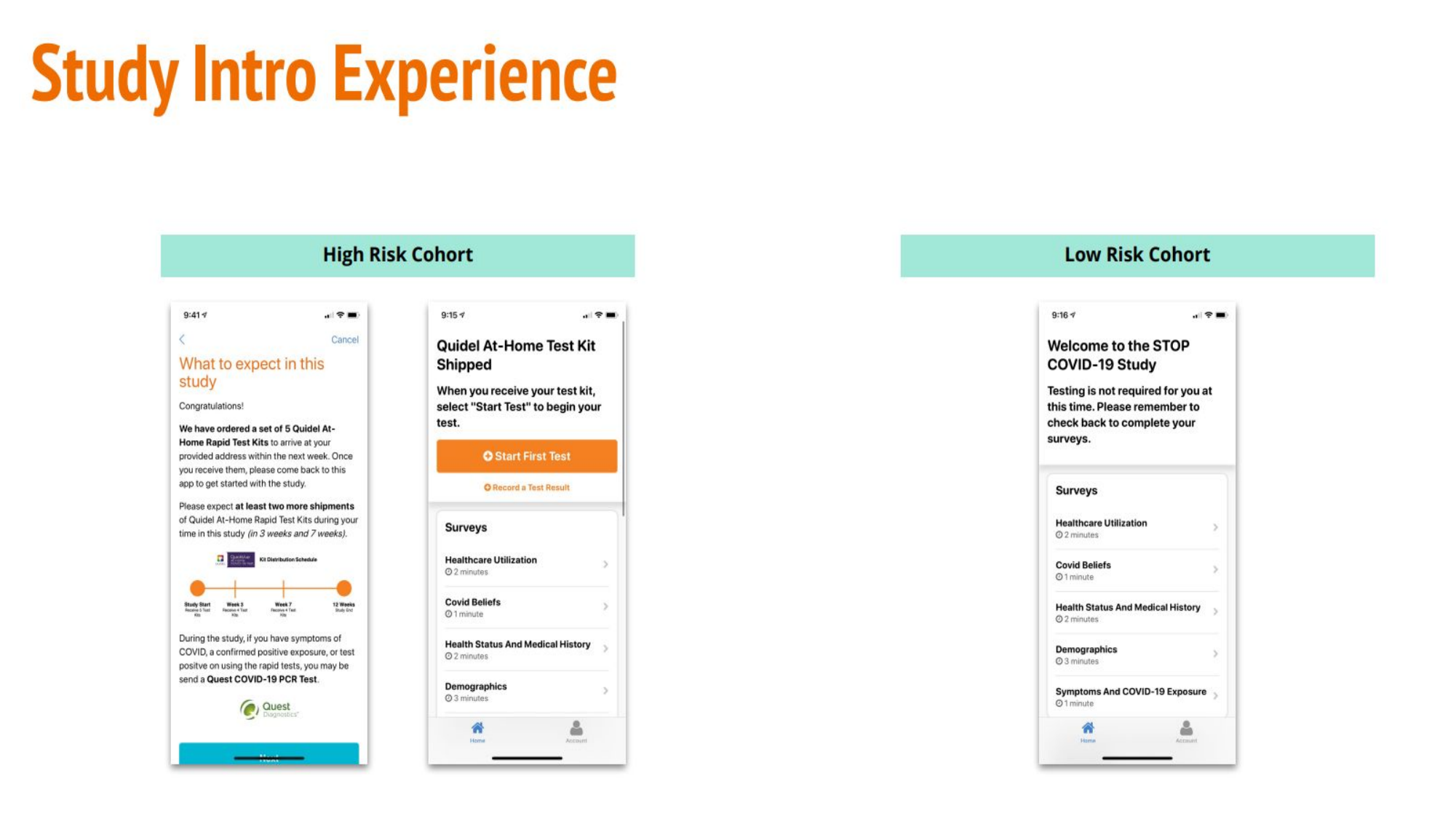

## Slide 3
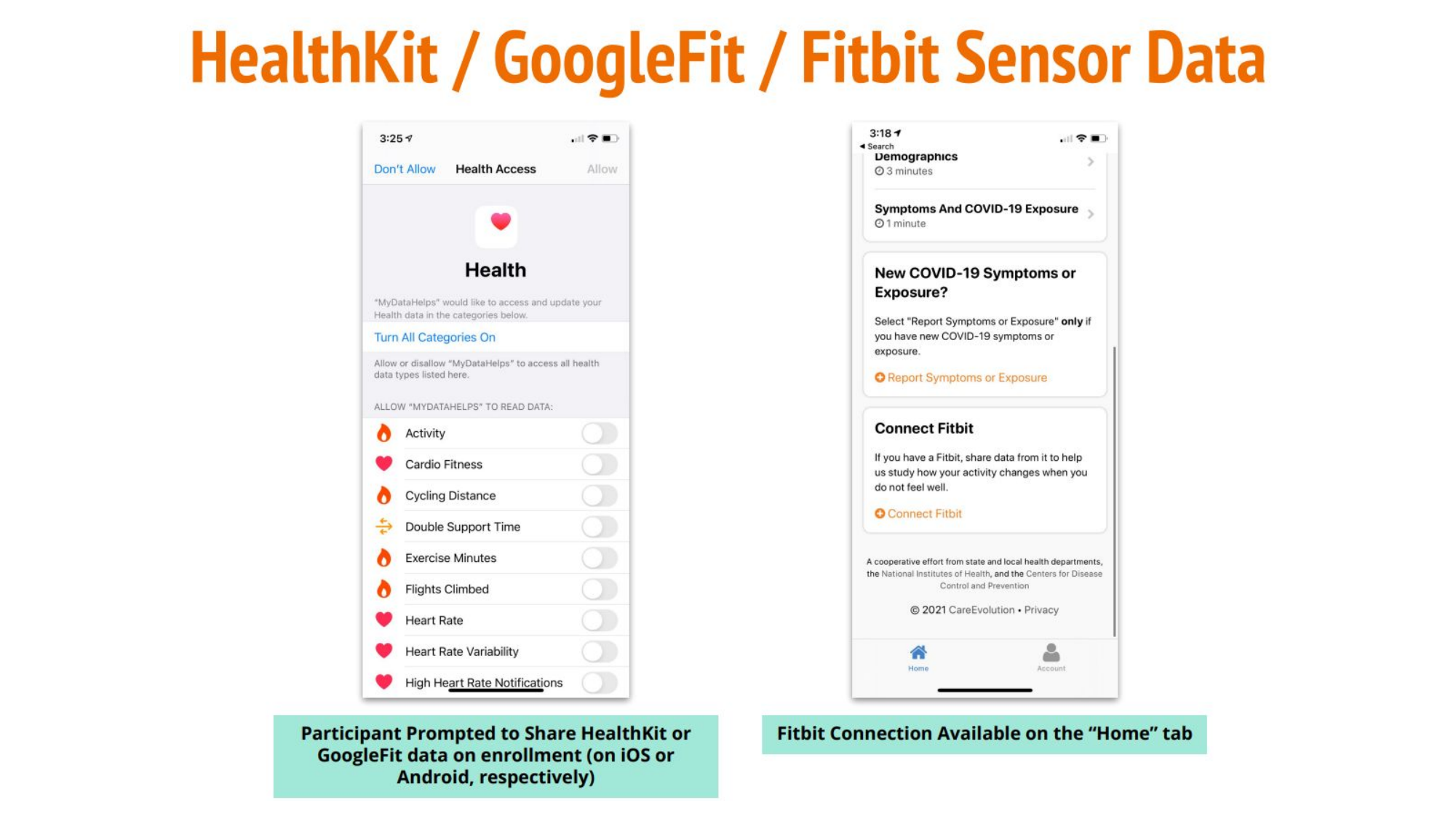

## Slide 4
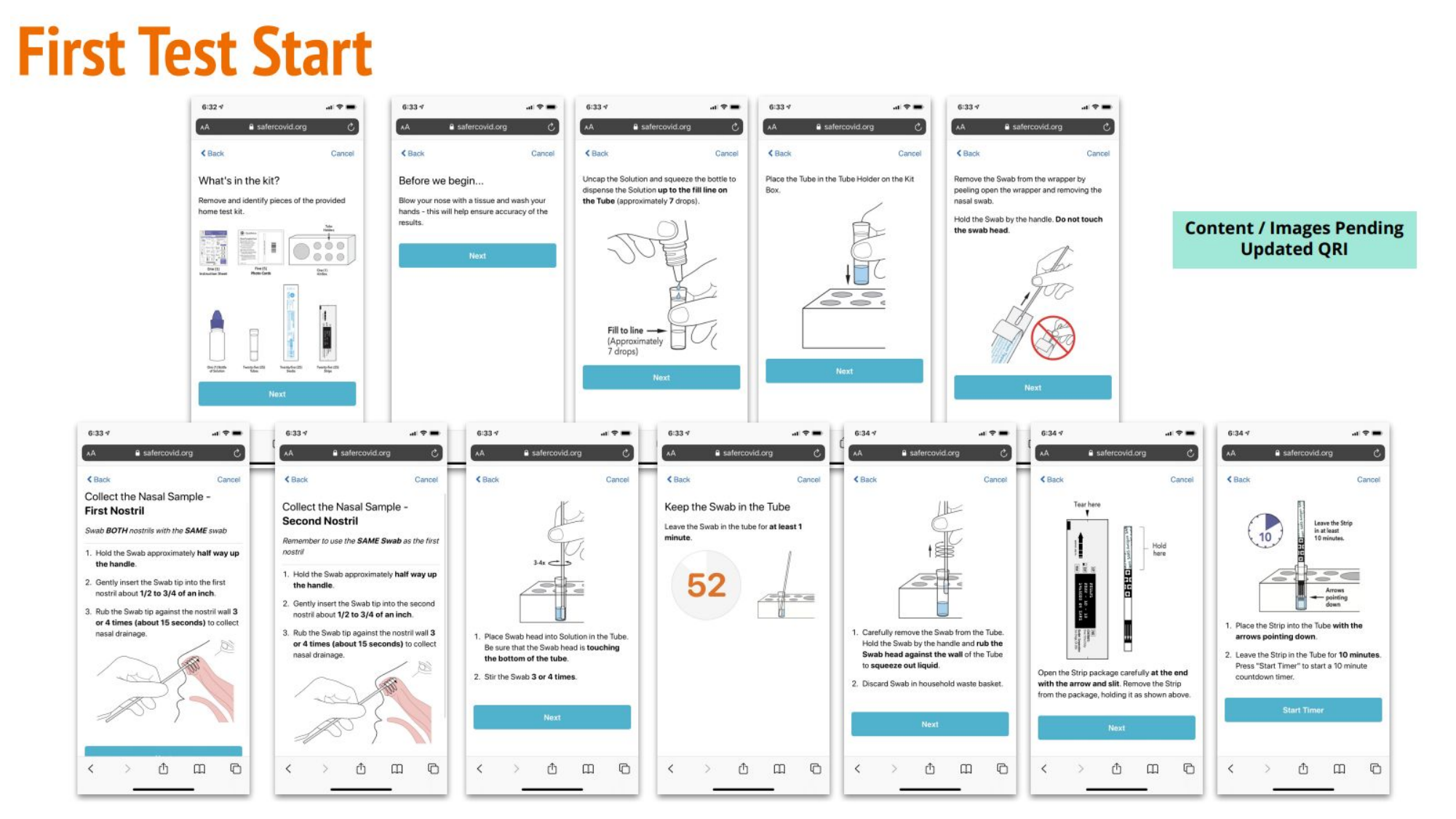

#

## Slide 5
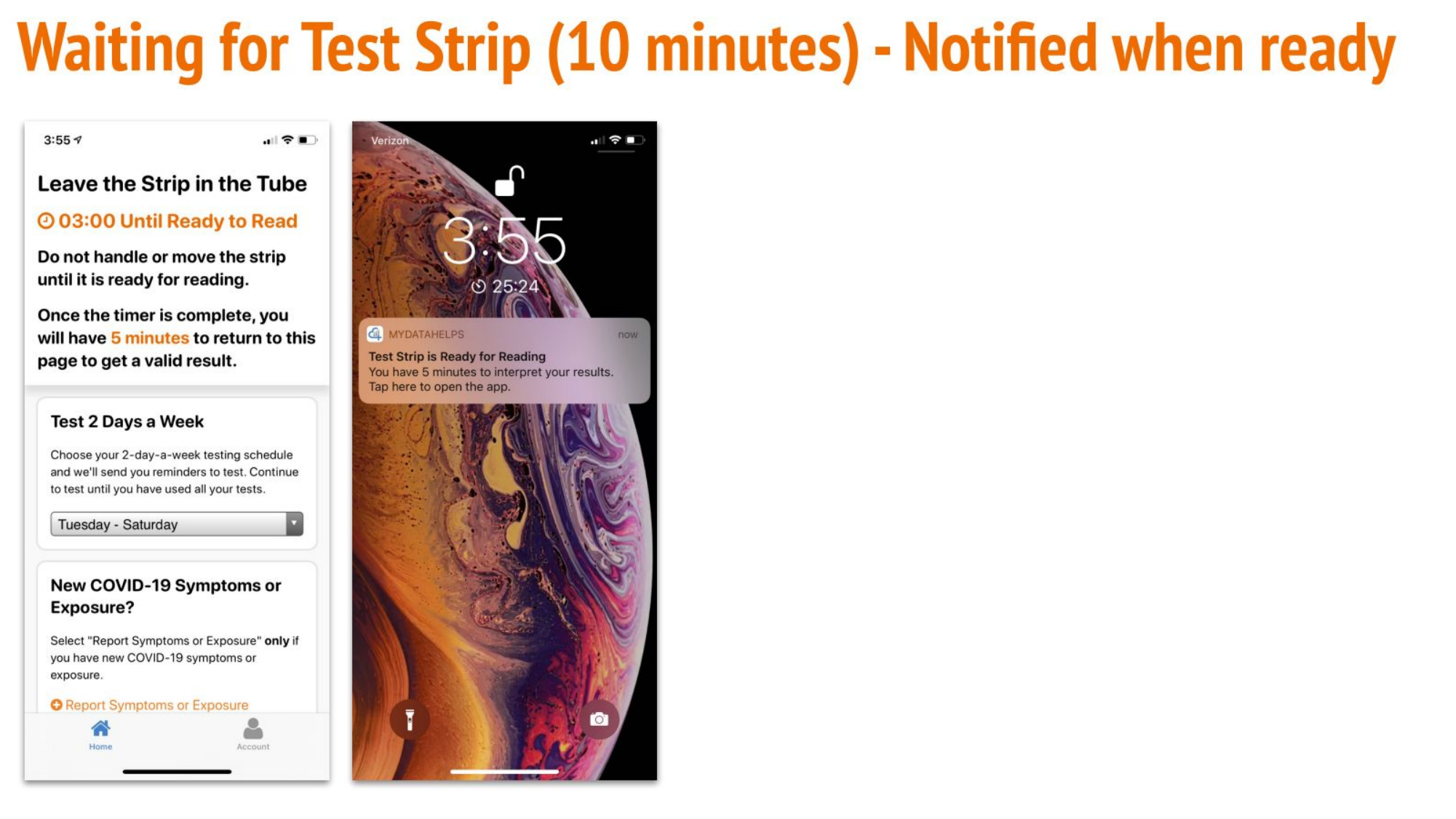

#

## Slide 6
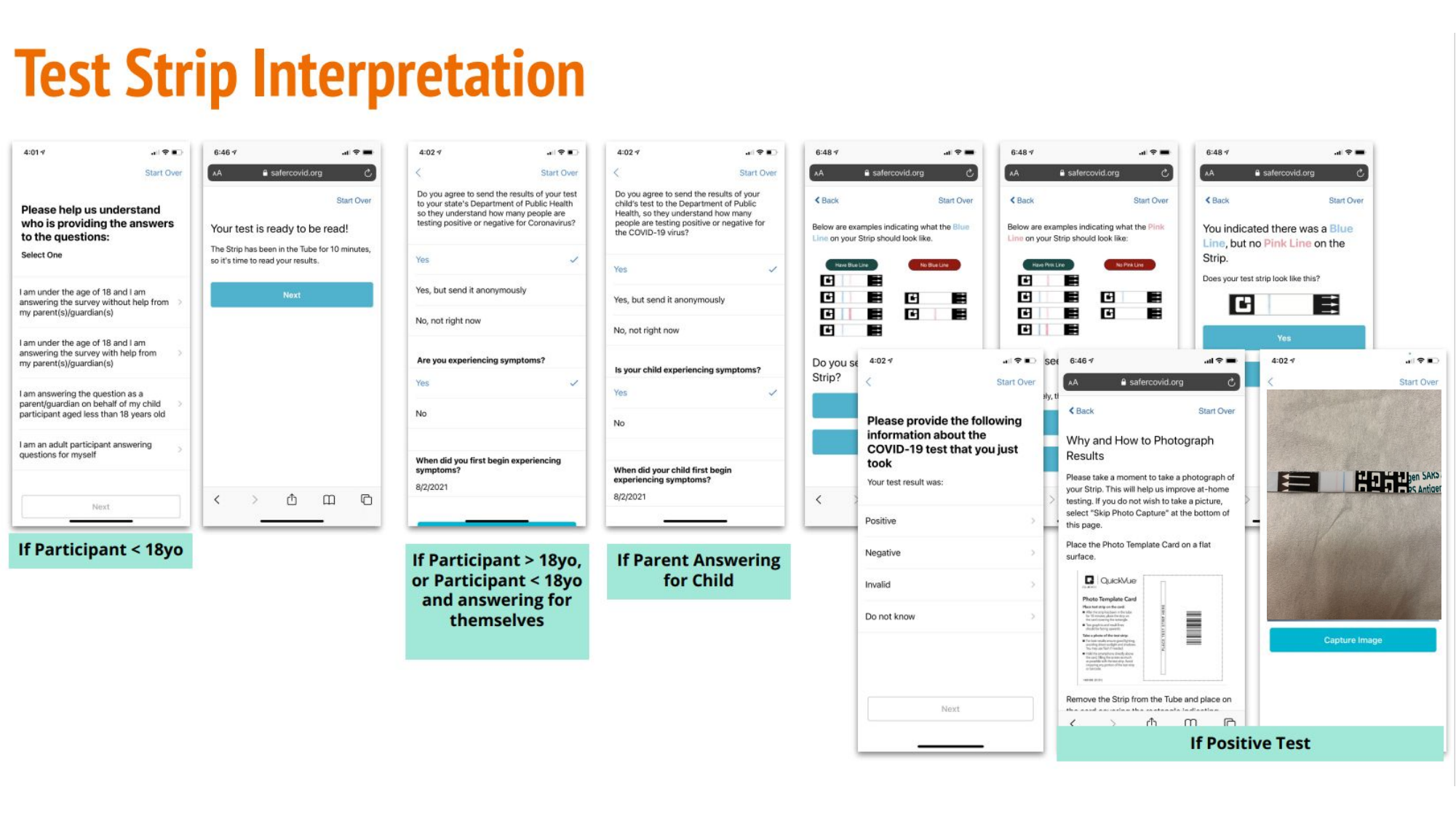

#

Supplement: Multimedia Appendix 1 [file formative_v6i6e38113_app1.pptx]
