# Supplementary material for: Design and Preliminary Findings of Adherence to the Self-Testing for Our Protection From COVID-19 (STOP COVID-19) Risk-Based Testing Protocol: Prospective Digital Study
Source: JMIR Form Res. 2022 Jun 16;6(6):e38113. doi: 10.2196/38113 (PMC9205422; doi:10.2196/38113)
Supplement: Multimedia Appendix 2 [file formative_v6i6e38113_app2.docx]

Multimedia Appendix 2. Supplemental tables and figures.

Table S1: Questionnaire Content and Timing

|  | Enrollment | Baseline | Post-testing Questionnaire | Weekly surveillance questionnaire | Exit Survey |
| --- | --- | --- | --- | --- | --- |
| Risk Stratification | X |  |  |  |  |
| Wearable Device Data Sharing | X |  |  |  |  |
| RADx Common Data Elements (Demographics) |  | X |  |  |  |
| Health Beliefs related to COVID-19 |  | X |  |  |  |
| Healthcare Utilization |  | X |  |  | X |
| Results Interpretation |  |  | X |  |  |
| Symptoms |  | X | X | X |  |
| Reporting of Test Results |  |  | X |  |  |
| Attitudes towards reporting test results |  |  | X |  |  |
| Close Contact to SARS-CoV-2 |  |  |  | X |  |
| App and Testing Experience |  |  |  |  | X |
| Usability/acceptability |  |  |  |  | X |

Figure S1: STOP COVID-19 Test Result Reporting Process


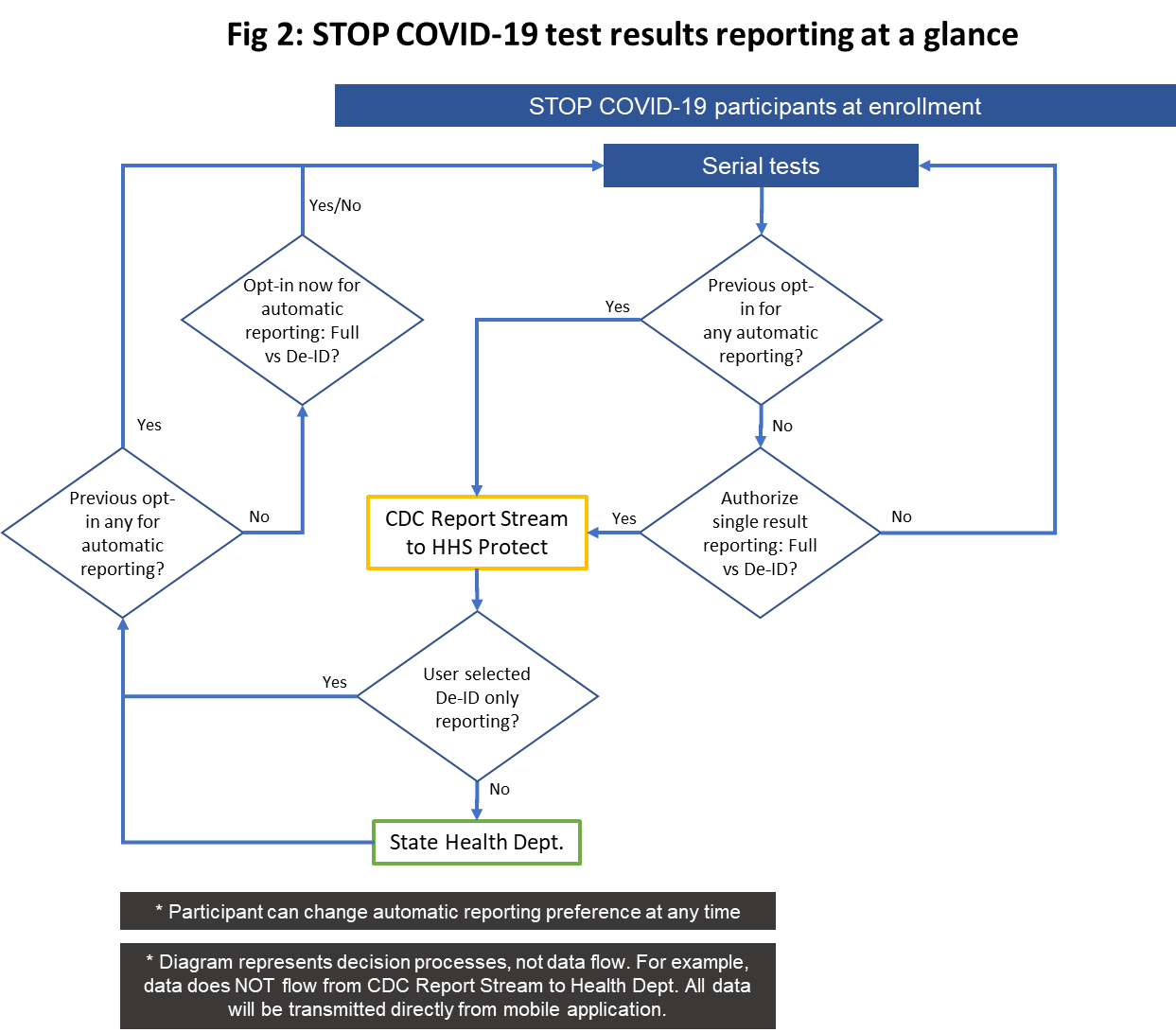


Figure S2: Current STOP COVID-19 Enrollment
